# Supplementary material for: Sorting at embryonic boundaries requires high heterotypic interfacial tension
Source: Nat Commun. 2017 Jul 31;8:157. doi: 10.1038/s41467-017-00146-x (PMC5537356; doi:10.1038/s41467-017-00146-x)
Supplement: Supplementary file 2 — Supplementary Software 1 [file 41467_2017_146_MOESM2_ESM.zip › PottsModel/SrcPottsModel/doc/model/class-use/EnergyFunction.Energy.html]

Uses of Class model.EnergyFunction.Energy


JavaScript is disabled on your browser.


Skip navigation links


- Overview
- Package
- Class
- Use
- Tree
- Deprecated
- Index
- Help

- Prev
- Next

- Frames
- No Frames

- All Classes

## Uses of Class model.EnergyFunction.Energy

- Packages that use EnergyFunction.Energy

  | Package | Description |
  | --- | --- |
  |  |  |
  | --- | --- |
  | engine |  |
  | model |  |
- - ### Uses of EnergyFunction.Energy in engine

    Methods in engine that return types with arguments of type EnergyFunction.Energy

    | Modifier and Type | Method and Description |
    | --- | --- |
    |  |  |
    | --- | --- |
    | `java.util.List<EnergyFunction.Energy>` | Utils.EnergyTracker.`getEnergyTypes()` |
    | `java.util.List<EnergyFunction.Energy>` | PottsEngine.`getEnergyTypes()` |

    Methods in engine with parameters of type EnergyFunction.Energy

    | Modifier and Type | Method and Description |
    | --- | --- |
    |  |  |
    | --- | --- |
    | `void` | Utils.EnergyTracker.`add(EnergyFunction.Energy t, double value)` |
    | `double` | Utils.EnergyTracker.`mean(EnergyFunction.Energy t)` |
    | `double` | Utils.EnergyTracker.`std(EnergyFunction.Energy t)` |

    Constructor parameters in engine with type arguments of type EnergyFunction.Energy

    Constructor and Description

    `EnergyTracker(java.util.List<EnergyFunction.Energy> energyTypes)`
  - ### Uses of EnergyFunction.Energy in model

    Methods in model that return EnergyFunction.Energy

    | Modifier and Type | Method and Description |
    | --- | --- |
    |  |  |
    | --- | --- |
    | `EnergyFunction.Energy` | PixelInteractionEnergyFunction.`getEnergyType()` |
    | `EnergyFunction.Energy` | PerimeterEnergyFunction.`getEnergyType()` |
    | `EnergyFunction.Energy` | InterfacialTensionEnergyFunction.`getEnergyType()` |
    | `EnergyFunction.Energy` | EnergyFunction.`getEnergyType()` |
    | `EnergyFunction.Energy` | AreaEnergyFunction.`getEnergyType()` |
    | `static EnergyFunction.Energy` | EnergyFunction.Energy.`valueOf(java.lang.String name)` Returns the enum constant of this type with the specified name. |
    | `static EnergyFunction.Energy[]` | EnergyFunction.Energy.`values()` Returns an array containing the constants of this enum type, in the order they are declared. |

    Methods in model that return types with arguments of type EnergyFunction.Energy

    | Modifier and Type | Method and Description |
    | --- | --- |
    |  |  |
    | --- | --- |
    | `java.util.List<EnergyFunction.Energy>` | EnergyManager.`getEnergyTypes()` |

Skip navigation links


- Overview
- Package
- Class
- Use
- Tree
- Deprecated
- Index
- Help

- Prev
- Next

- Frames
- No Frames

- All Classes
